# Effects of Bilateral Infraorbital and Infratrochlear Nerve Block on Emergence Agitation after Septorhinoplasty: A Randomized Controlled Trial

**DOI:** 10.3390/jcm8060769

**Published:** 2019-05-30

**Authors:** Hoon Choi, Seung Ho Jung, Jin Myung Hong, Young Ho Joo, Youme Kim, Sang Hyun Hong

**Affiliations:** 1 Deparment of Anesthesia and Pain Medicine, College of Medicine, The Catholic University of Korea, 222, Banpo-daero, Seocho-gu, Seoul 06591, Korea; hoonie83@naver.com (H.C.); ymkim0318@gmail.com (Y.K.); 2 Department of Anesthesia and Pain Medicine, College of Medicine, Yonsei University, 50-1, Yonsei-ro, Seodaemun-gu, Seoul 03722, Korea; bonobono1985@naver.com; 3 Department of Plastic Surgery, Dream Medical Group, 848, Nonhyeon-ro, Gannam-gu, Seoul 06022, Korea; hjm1985@naver.com; 4 Department of Otorhinolaryngology-Head & Neck Surgery, College of Medicine, Korea University, 73, Goryeodae-ro, Seongbuk-gu, Seoul 02841, Korea; akaboy@hanmail.net

**Keywords:** anesthesia, general, emergence delirium, pain, postoperative

## Abstract

Emergence agitation is common after septorhinoplasty, and postoperative pain is the main risk factor for this condition. Infraorbital and infratrochlear nerve block have been reported to facilitate pain management in patients after nasal procedures. The effect of peripheral nerve block on the incidence of emergence agitation has not been evaluated. Sixty-six patients that were scheduled for septorhinoplasty were assigned to receive bilateral infraorbital and infratrochlear nerve block with either 8 mL of 0.5% ropivacaine (Block group) or isotonic saline (Sham Block group). The incidence of emergence agitation was evaluated using the Riker sedation-agitation scale. Analgesic consumption, hemodynamic parameters, postoperative pain scores, adverse events, and patient satisfaction with analgesia were evaluated. The incidence of emergence agitation was lower in the Block group than in the Sham Block group (6 (20.0%) versus 20 (62.5%), *p* = 0.002). The mean intraoperative remifentanil consumption was lower in the Block group than in the Sham Block group (0.074 ± 0.014 μg/kg/min. versus 0.093 ± 0.019 μg/kg/min., respectively, *p* < 0.0001), as was the proportion of patients that needed postoperative tramadol administration and median postoperative pain score at 0–2 h after surgery (9 (30.0%) versus 21 (65.6%), *p* = 0.011; 3.0 (2.0–4.0) versus 4.0 (3.0–4.0), *p* < 0.0001, respectively). Hemodynamic parameters and the incidence of adverse events were similar between the two groups. The median patient satisfaction score with respect to analgesia was higher in the Block group than in the Sham Block group (3.5 (3.0–4.0) versus 3.0 (3.0–4.0), respectively, *p* = 0.034). The preoperative bilateral infraorbital and infratrochlear nerve block decreased the incidence of emergence agitation after septorhinoplasty.

## 1. Introduction

Emergence agitation (EA) is a postanesthetic condition, in which emergence from general anesthesia is accompanied by psychomotor features, such as agitation, confusion, disorientation, and violent behavior [1,2]. It can lead to serious complications for patients, including injury, hemorrhage, increased pain, self-extubation, and removal of catheters. EA can also cause harm to medical staff and may lead to increased hospital costs [3].

Although the etiology of EA is not well-understood, the known risk factors include age (young), sex (male), history of smoking, benzodiazepine premedication, sevoflurane anesthesia, high postoperative pain (defined as a pain score ≥ 4), and the presence of a tracheal and/or urinary catheter [1,4,5]. Ear, nose, and throat (ENT) surgery is associated with a high incidence of EA in both adults and children [2,4,5]. The incidence of EA after ENT surgery can reach up to 55.4% in adults, although it is more common in the pediatric population [4]. However, previous studies have focused on children rather than adults, and preventive measures for EA in adults have not been investigated in detail.

Pain management can be an effective preventive measure for EA. After septorhinoplasty, the patients experience a high level of postoperative pain [6,7]. Therefore, ultimodal pain management, including peripheral nerve block, is recommended in these patients [8,9]. The infraorbital nerve innervates the skin of the nose and the septum mobile nasi, while the infratrochlear nerve innervates the root of the nose [10,11]. The peripheral nerve block of these two nerves has been reported to facilitate pain management in patients after nasal procedures [12,13,14,15,16].

In this study, we evaluated the hypothesis that pain management through preoperative bilateral infraorbital and infratrochlear nerve block reduces the incidence of EA in adult patients after septorhinoplasty.

## 2. Materials and Methods

The Institutional Review Board of The Armed Forces Medical Command, Seongnam-si, Korea (approval number AFMC-16-IRB-028; date of approval by the ethics committee 8 April 2016) approved this study was approved, and it was registered at ClinicalTrials.gov (Identifier NCT02751268). From April 2016 to March 2017, we enrolled patients aged 18–65 years with American Society of Anesthesiologists (ASA) physical status I–II, and who were scheduled for septorhinoplasty under general anesthesia. The exclusion criteria were as follows: ASA physical status ≥ III, body mass index (BMI) > 35 kg/m^2^, known allergy to local anesthetic drugs, preoperative chronic pain, proven coagulopathy, and inability to provide informed consent. Written informed consent was obtained from all of the patients before randomization.

The patients were randomly assigned to one of two groups by a single nurse, who was not involved in the anesthetic management of the patient, while using a computer-generated randomization sequence with random block sizes of two and four [17]. The same nurse prepared syringes containing the nerve block solution. Syringes for the block group (Block group) were filled with 8 mL of 0.5% ropivacaine (Ropiva®, Hanlim, Seoul, Korea), and the syringes for the control group (Sham Block group) were filled with 8 ml of 0.9% saline for a sham block. The patient, surgeon, anesthesiologist, and investigator were all blinded to the study solution and the adequacy of the block.

**Figure 1:**
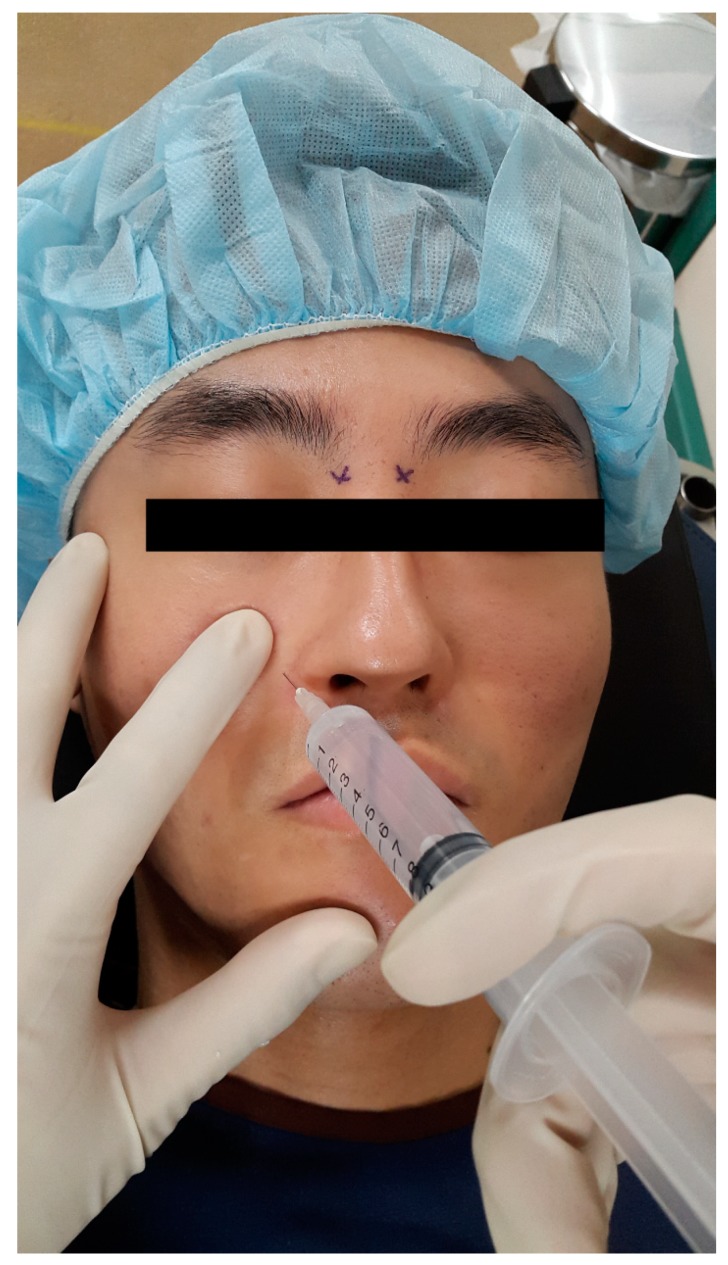
Infraorbital nerve block. The index finger of the non-dominant hand was positioned above the infraorbital foramen, and the needle was advanced until it was felt beneath the finger to avoid globe penetration injury.

**Figure 2:**
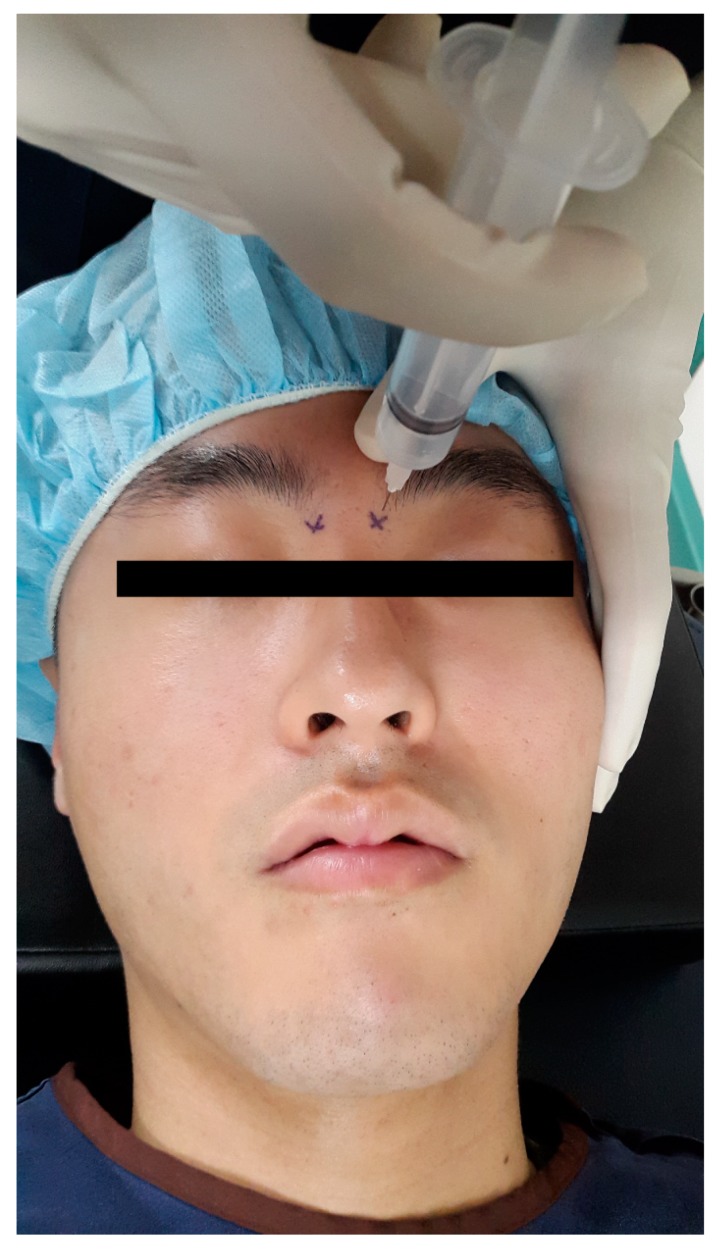
Infratrochlear nerve block.

Thirty minutes prior to surgery, all of the patients received bilateral infraorbital and infratrochlear nerve block in the preoperative waiting room. The patient’s head was positioned on a central line in the supine position. Infraorbital nerve block was performed while using an extraoral approach [18]. A 25 gauge needle was inserted laterally to the ipsilateral nostril after palpating the infraorbital ridge to locate the infraorbital foramen. The index finger of the non-dominant hand was positioned above the infraorbital foramen, and the needle was advanced until it was felt beneath the finger to avoid globe penetration injury [19]. 3 mL of the study solution was slowly injected after negative aspiration of blood was confirmed, taking care not to inject into the foramen itself (Figure 1). Inserting the needle 1 cm above the inner canthus, targeting the junction of the orbit and the nasal bone, performed Infratrochlear nerve block. After negative aspiration of blood, 1 mL of the study solution was injected (Figure 2) [10,11]. Contralateral nerve block was performed in the same manner. Pressure was applied to four injection points for one minute to prevent hematoma. Before leaving the preoperative waiting room, pinprick and cold sensation tests were performed by a nurse who did not participate in patient randomization, syringe preparation, the anesthetic management of the patient, and outcome assessment, and the block was considered to be inadequate if pain or cold sensations were felt on the skin of the nose. The results were recorded on a separate result sheet to enable blinding.

Upon arrival in the operating room, all of the patients received intravenous (IV) dexamethasone 5 mg premedication for the prevention of postoperative nausea and vomiting (PONV) and surgery-related edema and ecchymosis [20]. Routine monitoring, including electrocardiography, pulse oximetry, non-invasive blood pressure, and end-tidal CO_2_ (ETCO_2_), were applied to the patients and recorded every five minutes. Anesthesia was induced with IV propofol 2–2.5 mg/kg. IV rocuronium 0.6 mg/kg and IV remifentanil 0.5–1 μg/kg/min was given to facilitate tracheal intubation with a cuffed tube. After tracheal intubation, mechanical ventilation was initiated with a mixture of 60–70% air and 30–40% oxygen, and minute ventilation was adjusted to maintain ETCO_2_ between 34 and 39 mmHg. Anesthesia was maintained with sevoflurane (1.0–1.5 age-adjusted minimal alveolar concentration) and the continuous infusion of IV remifentanil 0.05–0.2 μg/kg/min to maintain the bispectral index (BIS) (A-2000TM SP; Aspect Medical Systems, Norwood, MA, USA) values between 40–60 and minimize bleeding in the area of operation.

After the induction of anesthesia, the surgical site, including nasal septum, nasal turbinate, columella, and nasal dorsum, was infiltrated with a 10 mL mixture of 1% lidocaine and 10 μg/mL epinephrine for local anesthesia and intraoperative bleeding reduction [20,21]. The surgical procedure was performed while using an open septorhinoplasty approach. At the end of surgery, nasal packing was carried out using a polyvinyl alcohol sponge (Merocel®; Medtronic Inc., Minneapolis, MN, USA), and Joseph dressings with splints (Aquasplint; Aquaplast Inc., Wyckoff, NJ, USA) were applied [20].

Thirty minutes before the end of the surgery, all of the patients received IV paracetamol 1 g for postoperative pain management and IV ramosetron 0.3 mg for PONV prophylaxis. At the end of surgery, oral suction was performed, and IV glycopyrrolate 0.004 mg/kg and IV neostigmine 0.02 mg/kg were administered to all patients for neuromuscular reversal. To ensure the smooth emergence, all stimuli except repeated verbal requests to open the eyes were prevented. Tracheal extubation was performed after confirming spontaneous ventilation, response to verbal commands and a BIS value ≥ 80, and the patients were then sent to the post-anesthesia care unit (PACU).

EA was assessed while using the Riker Sedation-Agitation Scale (SAS): 1 =  minimal or no response to noxious stimuli; 2 = arousal to physical stimuli but noncommunicative; 3 = difficult to arouse but awakens to verbal stimuli or gentle shaking; 4  =  calm and follows commands; 5 =  anxious or physically agitated but calm in response to verbal instructions; 6 = requires restraint and frequent verbal reminding of limits; and, 7  = attempting to remove tracheal tube or catheters or striking staff [22]. The patients were continuously monitored from the time of extubation until discharge from the PACU by the investigator, who recorded the SAS score whenever it changed. The highest SAS score during this time period was used for the assessment of EA. SAS scores ≥5 were defined as EA, and SAS scores >5 were defined as severe EA. When the Aldrete recovery score was ≥9, the patients were discharged from the PACU.

Postoperative pain was assessed while using a numerical rating scale (NRS) that ranged from 0 (no pain) to 10 (maximum pain imaginable). For patients with NRS scores > 3, IV tramadol 25 mg was administered at 10-min intervals. If pain persisted after two administrations of IV tramadol, IV pethidine 12.5 mg was administered at 10-min intervals. The patients’ highest NRS score was recorded at 0–2 h, 2–8 h, 8–24 h, and 24–48 h after arrival at the PACU (0 h).

Neurological deficits in the blocked area, such as paresthesia and/or prolonged paralysis of the upper lip, injection site edema, hematoma, or PONV, were recorded as adverse events and they were evaluated at the PACU and 24 h after the surgery. Patient satisfaction with the quality of pain management was assessed on the second postoperative day using the following scale: 1= very unsatisfactory; 2 = rather unsatisfactory; 3 = fair; 4 = rather satisfactory; 5 = very satisfactory.

The sample size was calculated based on the incidence of EA. According to a pilot study that was performed in our institution, bilateral infraorbital and infratrochlear nerve block decreased the incidence of EA from 60% to 20% (unpublished data). To achieve a power of 80% at a significance level of 0.05, 30 subjects were required in each group. 33 subjects were included in each group when assuming an approximate 10% dropout rate.

Statistical analyses were performed using SPSS software (ver.20.0; SPSS, Inc., Chicago, IL, USA). The normality of distribution was verified using the Shapiro–Wilk test. Continuous variables were compared while using Student’s *t*-test or the Mann–Whitney U test, depending on the normality of the data, and the categorical variables were compared using Fischer’s exact test or the χ2 test, as appropriate. Continuous variables were compared using repeated measures ANOVA with Bonferroni correction. *p* values < 0.05 was considered to be statistically significant. All of the values were expressed as mean ± standard deviation, median (IQR), or number (proportion). 

## 3. Results

**Figure 3:**
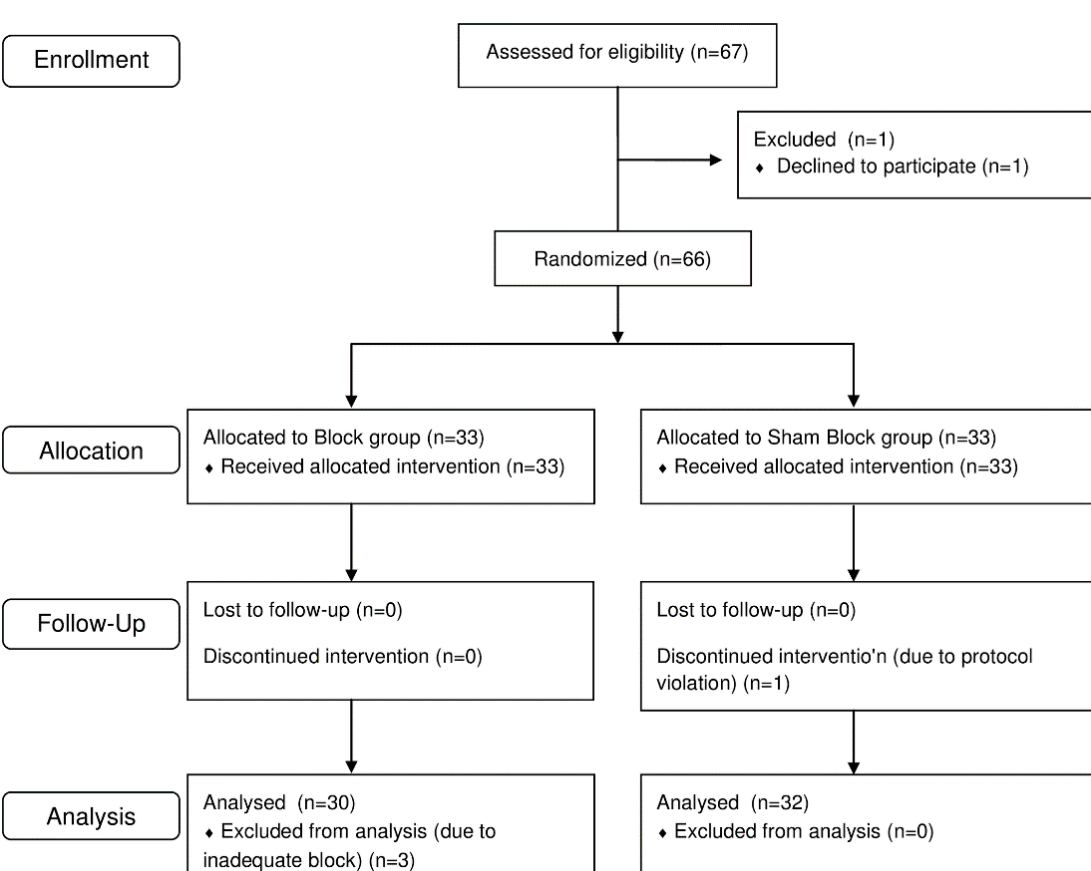
CONSORT flowchart of the study.

In total, 67 patients were assessed for eligibility and 66 were enrolled in the study. Four patients were excluded: three in the Block group due to inadequate block and one in the Sham Block group, owing to protocol violation (the patient received a rescue analgesic not specified in the protocol). For the remaining 62 patients, no data or measurements were missing or lost. Figure 3 presents the study flow chart. The patient characteristics were similar in both groups (Table 1).

The mean duration of the surgery and anesthesia were similar in both groups (95.5 ± 15.3 min in the Block group versus 99.6 ± 14.5 min in the Sham Block group, *p* = 0.288; and, 113.7 ± 15.5 min in the Block group versus 120.3 ± 15.3 min in the Sham Block group, *p* = 0.093, respectively). Mean intraoperative mean arterial blood pressure (MBP) and heart rate (HR) at baseline, time of incision, duration of surgery, and time of skin closure were all similar in both of the groups (*p* = 0.258 and *p* = 0.670, respectively). The mean intraoperative remifentanil consumption, defined as total remifentanil used during the surgery (μg) divided by the patient’s weight (kg) and the duration of anesthesia (min), was lower in the Block group compared to the Sham Block group (0.074 ± 0.014 μg/kg/min versus 0.093 ± 0.019 μg/kg/min, respectively, *p* < 0.0001) (Table 1).

**Figure 4:**
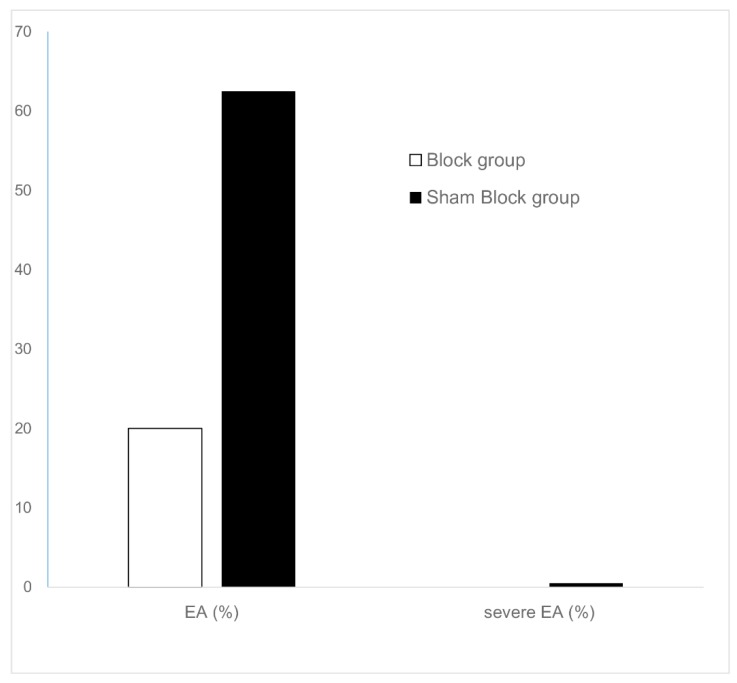
Incidence of emergence agitation (white) and severe emergence agitation (black).

The incidence of EA was lower in the Block group as compared to the Sham Block group (six (20.0%) in the Block group versus 20 (62.5%) in the Sham Block group, *p* = 0.002). One patient in the Sham Block group exhibited severe EA, while no patients in the Block group exhibited severe EA (Figure 4).

The proportion of patients that needed tramadol administration after surgery was lower in the Block group when compared to the Sham Block group (9 (30.0%) versus 21 (65.6%), respectively, *p* = 0.011). No difference was observed in pethidine administration between the two groups. The median NRS score of patients at 0–2 h was lower in the Block group when compared to the Sham Block group (3.0 (2.0–4.0) and 4.0 (3.0–4.0)), respectively, *p* < 0.0001), but no differences were found at later time intervals. No patient in either group showed any neurological deficits. The two groups had similar incidences of edema, hematoma, and PONV at the PACU and 24 h after the surgery. The median patient satisfaction score for pain management on the second postoperative day was lower in the Block group as compared to the Sham Block group (3.5 (3.0–4.0) versus 3.0 (3.0–4.0), respectively, *p* < 0.034) (Table 2).

We performed a risk assessment to investigate the correlation between the pain score at 0–2 h after surgery and EA. NRS scores > 3 at 0–2 h after the surgery and sham block treatment were related to the incidence of EA (odds ratio 16.50, *p* < 0.0001 and odds ratio 6.67, *p* = 0.001, respectively). However, a history of smoking was not related to the incidence of EA (odds ratio 2.12, *p* = 0.189) (Table 3).

## 4. Discussion

The results of this study demonstrate that pain management through bilateral infraorbital and infratrochlear nerve block decreases the incidence of EA in patients after septorhinoplasty. Although intraoperative hemodynamic parameters and postoperative adverse events were similar in both of the groups; patients who received the block had lower pain scores 0–2 h after the surgery and higher patient satisfaction scores.

The reported incidence of EA is more than 20% in adult patients after general anesthesia [1,5]. The known risk factors for EA include age (young), sex (male), history of smoking, benzodiazepine premedication, sevoflurane anesthesia, high postoperative pain score, and the presence of tracheal and/or urinary catheters [4,5]. Patients undergoing ENT procedures are at greater risk for EA than other patients; among ENT patients, the incidence of EA is 55.4% [4]. The patients that enrolled in the present study were therefore expected to exhibit a high incidence of EA. Owing to the nature of the military hospital, all of the patients were young males. All patients received sevoflurane anesthesia, and septorhinoplasty is related to high postoperative pain scores [6,7]. As expected, the incidence of EA in Group B was 62.5%, which was higher than the previously reported results [4,5].

Our risk assessment demonstrated a strong correlation between pain and EA, as expected. On the other hand, a history of smoking was not related to the incidence of EA in our study, which was probably owing to the small sample size. It has recently been suggested that methods are required for distinguishing between EA and agitation due to pain in pediatric patients [23]. Young children are not capable of reporting their pain, and EA can therefore be mistaken for agitation due to pain in such cases. However, the same is not true for adults, who, in most cases, do not become agitated, simply because they cannot express pain.

Analgesic interventions that reduce 1 or more points in pain scores indicates a clinically important improvement, and a pain score of 3.3 or less indicates acceptable pain control after surgery [24]. Therefore, from the results of this study, we believe that there is a clinically important improvement regarding pain in the Block group when compared to the Sham block group. Moreover, the Block group showed acceptable pain control throughout the entire postoperative period. 

To date, most of the attempts to prevent EA have focused on pharmacological methods. In children, opioids [25], ketamine [26], dexmedetomidine [27,28], propofol [29], and antiepileptic medications [30,31] have been extensively studied for the prevention of EA with good results. Ketamine [32], dexmedetomidine [33], and propofol [34] were shown to effectively reduce EA in adults. However, pharmacological prevention can cause residual sedation and hemodynamic changes, such as hypotension, hypertension, and bradycardia, resulting in prolonged PACU stay and questionable cost-effectiveness. In our study, we focused on pain management through peripheral nerve block; therefore, our patients did not experience any residual sedation or hemodynamic changes.

Previous attempts to reduce the incidence of EA through regional and peripheral nerve block were only targeted at pediatric populations [35,36,37]. The patients in our study were all adults, who are also susceptible to EA. A previous study evaluating infraorbital nerve block in children undergoing cleft lip surgery found that this intervention resulted in a reduction in EA, from 42% to 16% [37]. Our study reduced EA from 62.5% to 20.0%, an effect of similar magnitude.

Multimodal pain management therapy with systemic analgesics and peripheral nerve block is recommended for septorhinoplasty whenever possible [8,9]. In the current study, we administered IV paracetamol to both of the groups before emergence, and IV tramadol and pethidine were administered as rescue analgesics. The pain score in the Sham block group in our study was consistent with pain scores in previous studies with septorhinoplasty without peripheral nerve blocks [7,12]. The facial nerves innervating areas within the surgical field of septorhinoplasty include the infraorbital and infratrochlear nerves [10,11]. The infraorbital nerve is a branch of the maxillary division of the trigeminal nerve, and it supplies sensation to the skin of the nose and the septum mobile nasi. The infratrochlear nerve is an extraconal branch of the nasociliary nerve, a branch of the ophthalmic division of the trigeminal nerve, and it supplies sensation to the root of the nose. The bilateral infraorbital nerve block has previously been reported to decrease postoperative pain scores [13,14,15,16]. Bilateral infratrochlear nerve block, in combination with infraorbital nerve block, also resulted in decreased pain scores [12]. However, previous studies performed peripheral nerve block after general anesthesia, and the success of the block was assessed based on postoperative pain scores and the dose of analgesics during or after surgery. In our study, we performed nerve blocks prior to the induction of anesthesia; therefore, we were able to confirm the adequacy of the block while using pinprick and cold sensation tests. Successful block was observed in 90.9% of patients (30 out of 33), and the results for the patients with failed blocks were not included in the analysis. The percentage of patients who reported NRS values > 3 was 30% in the present study, which was lower than the 47% that was reported in a previous study while using the same peripheral nerve block [12].

There were several limitations to the current study. First, because the etiology of EA is unclear, factors other than pain might have resulted in EA. There are conflicting results on the risk factors of EA, especially regarding anxiety, gender, and nasal packing in ENT surgery [1,4,5]. Second, the patients who participated in our study were all young males and were therefore not representative of the general population. However, the study subjects do represent a group at high risk of EA, for which active intervention is especially important. Therefore, the results of the present study may overestimate the incidence of EA and the effects of pain management through peripheral nerve block. Third, the SAS scoring system that we adopted in this study was not designed for the evaluation of EA. Currently, the SAS and the Richmond Sedation-Agitation Scale are the two most widely accepted scales of agitation. These two scales were created for patients in intensive care and both have excellent inter-rater reliability [22,38,39]. A scoring system that was solely established for the PACU does not exist, and therefore we used the SAS, like in previous studies [1,33,40]. Fourth, the blinding of the patients to the study might have been incomplete, since the block was performed and tested preoperatively. However, this is the only possible method in order to accurately test the adequacy of the block, which is adapted by majority of trials on preoperative blocks for analgesia. Fifth, although sevoflurane is considered to be a risk factor for EA, we did not compare administered or end-tidal sevoflurane between the two groups. However, we induced hypnosis with sevoflurane, as guided by BIS and achieved adequate analgesia with remifentanil, as guided by hemodynamics. Indeed, remifentanil consumption during the surgery was lower in the block group when compared to the sham block group. Additionally, sevoflurane as a risk factor for EA has been studied, not by dosing, but only as its use. Finally, alternative methods for pain management using peripheral nerve block in septorhinoplasty require further investigation. The two nerve blocks that were performed in this study do not cover all the regions of surgery; nerve blocks at a more proximal level, such as maxillary nerve block and nasociliary nerve block, were not used due to the difficulty of application and the need of neurostimulation [10,11]. Furthermore, the use of ultrasound guidance may increase the efficacy and safety of the procedure [41].

In conclusion, pain management through preoperative bilateral infraorbital and infratrochlear block reduced the EA in septorhinoplasty patients after surgery. The peripheral nerve block facilitated postoperative pain management in patients after septorhinoplasty. Further studies are needed to determine the benefits of the peripheral and regional nerve block over EA in other surgeries.

## Figures and Tables

**Table 1:** Preoperative and intraoperative characteristics. The data are presented as the mean ± standard deviation, median (IQR) and number (proportion; %). NRS, numerical rating scale; PACU, post-anesthesia care unit; PONV, postoperative nausea and vomiting.

	Block Group (*n* = 30)	Sham Block Group (*n* = 32)	*p* Value
**Age (year)**	21.97 ± 1.474	22.38 ± 3.077	0.512
**Height (cm)**	174.5 ± 5.1	175.6 ± 5.6	0.411
**Weight (kg)**	69.8 ± 8.2	69.9 ± 8.3	0.935
**BMI (kg/m^2^)**	22.9 ± 2.3	22.6 ± 2.2	0.659
**History of smoking**	18 (60.0%)	24 (75.0%)	0.322
**ASA Class**			0.072
I	20 (66.7%)	13 (40.6%)	
II	10 (33.3%)	19 (59.4%)	
**Duration of surgery (min)**	95.5 ± 15.3	99.6 ± 14.5	0.288
**Duration of anesthesia (min)**	113.7 ± 15.5	120.3 ± 15.3	0.093
**Remifentanil consumption** **(μg/kg/min)**	0.074 ± 0.014	0.093 ± 0.019	**<0.0001**
**Mean blood pressure (mmHg)**			0.258
Baseline	97.0 ± 6.7	93.4 ± 7.4	0.051
Skin incision	83.1 ± 7.2	84.6 ± 10.2	0.518
During surgery	74.1 ± 6.7	72.6 ± 6.2	0.370
Skin closure	76.9 ± 7.7	74.4 ± 8.1	0.227
**Heart rate (beats/min)**			0.670
Baseline	71.2 ± 9.4	70.1 ± 9.8	0.661
Skin incision	67.9 ± 9.2	70.7 ± 11.3	0.302
During surgery	63.4 ± 8.7	62.1 ± 6.5	0.484
Skin closure	66.6 ± 8.5	63.4 ± 7.0	0.109

**Table 2:** Recovery characteristics. The data are presented as the mean ± standard deviation, median (IQR) and number (proportion; %). NRS, numerical rating scale; PACU, post-anesthesia care unit; PONV, postoperative nausea and vomiting.

	Block Group (*n* = 30)	Sham Block Group (*n* = 32)	*p* Value
**Tramadol use**	9 (30.0%)	21 (65.6%)	**0.011**
**Pethidine use**	0 (0.0%)	2 (6.2%)	0.501
**Pain score (in NRS)**			**0.003**
0–2 h	3.0 (2.0–4.0)	4.0 (3.0–4.0)	**<0.000** **1**
2–8 h	2.0 (2.0–2.0)	2.0 (2.0–3.0)	0.680
8–24 h	1.0 (1.0–2.0)	1.0 (1.0–2.0)	0.384
24–48 h	0.0 (0.0–1.0)	0.5 (0.0–1.0)	0.256
**Adverse events**			
Neurologic deficit in PACU	0	0	
Neurologic deficit after 24 h	0	0	
Edema in PACU	4 (13.3%)	5 (15.6%)	1.000
Edema after 24 h	3 (10.0%)	4 (12.5%)	1.000
Hematoma in PACU	3 (10.0%)	3 (9.4%)	1.000
Hematoma after 24 h	3 (10.0%)	2 (6.3%)	0.940
PONV in PACU	4 (13.3%)	4 (12.5%)	1.000
PONV after 24 h	1 (3.3%)	0 (0.0%)	0.974
**Patient satisfaction score**	3.5 (3.0–4.0)	3.0 (3.0–4.0)	**0.034**

**Table 3:** Risk assessment for emergence agitation. NRS, numerical rating scale.

	Odds Ratio	Confidence Interval	*p* Value
**NRS > 3 at 0–2 h**	16.50	4.47–60.87	**<0.000** **1**
**Received sham block**	6.67	2.12–20.96	**0.001**
**History of smoking**	2.12	0.68–6.58	0.189
